# Profiling circulating microRNA expression in a mouse model of nerve allotransplantation

**DOI:** 10.1186/1423-0127-20-64

**Published:** 2013-09-05

**Authors:** Cheng-Shyuan Rau, Johnson Chia-Shen Yang, Shao-Chun Wu, Yi-Chun Chen, Tsu-Hsiang Lu, Ming-Wei Lin, Yi-Chan Wu, Siou-Ling Tzeng, Chia-Jung Wu, Ching-Hua Hsieh

**Affiliations:** 1 Department of Neurosurgery, Kaohsiung Chang Gung Memorial Hospital and Chang Gung University College of Medicine, No. 123, Ta-Pei Road, Kaohsiung City, Niao-Sung District, 833, Taiwan; 2 Department of Plastic and Reconstructive Surgery, Kaohsiung Chang Gung Memorial Hospital and Chang Gung University College of Medicine, Center for Vascularized Composite Allotransplantation, No. 123, Ta-Pei Road, Kaohsiung City, Niao-Sung District, 833, Taiwan; 3 Department of Anesthesiology, Kaohsiung Chang Gung Memorial Hospital and Chang Gung University College of Medicine, No. 123, Ta-Pei Road, Kaohsiung City, Niao-Sung District, 833, Taiwan

**Keywords:** microRNAs (miRNAs), Circulating microRNAs, Nerve allotransplantation, FK506

## Abstract

**Background:**

The lack of noninvasive biomarkers of rejection remains a challenge in the accurate monitoring of deeply buried nerve allografts and precludes optimization of therapeutic intervention. This study aimed to establish the expression profile of circulating microRNAs (miRNAs) during nerve allotransplantation with or without immunosuppression.

**Results:**

Balb/c mice were randomized into 3 experimental groups, that is, (1) untreated isograft (Balb/c → Balb/c), (2) untreated allograft (C57BL/6 → Balb/c), and (3) allograft (C57BL/6 → Balb/c) with FK506 immunosuppression. A 1-cm Balb/c or C57BL/6 donor sciatic nerve graft was transplanted into sciatic nerve gaps created in recipient mice. At 1, 3, 7, 10, and 14 d after nerve transplantation, nerve grafts, whole blood, and sera were obtained for miRNA expression analysis with an miRNA array and subsequent validation with quantitative real-time PCR (qRT-PCR). Three circulating miRNAs (miR-320, miR-762, and miR-423-5p) were identified in the whole blood and serum of the mice receiving an allograft with FK506 immunosuppression, within 2 weeks after nerve allotransplantation. However, these 3 circulating miRNAs were not expressed in the nerve grafts. The expression of all these 3 upregulated circulating miRNAs significantly decreased at 2, 4, and 6 d after discontinuation of FK506 immunosuppression. In the nerve graft, miR-125-3b and miR-672 were significantly upregulated in the mice that received an allograft with FK506 only at 7 d after nerve allotransplantation.

**Conclusions:**

We identified the circulating miR-320, miR-762, and miR-423-5p as potential biomarkers for monitoring the immunosuppression status of the nerve allograft. However, further research is required to investigate the mechanism behind the dysregulation of these markers and to evaluate their prognostic value in nerve allotransplantation.

## Background

Nerve allograft transplantation is now a clinical reality for select patients who sustain a considerable nerve injury with nerve defect [1]. During nerve regeneration through the allograft, temporary immunosuppression is required for the nerve allotransplantation to be successful. Cyclosporin A has been used to increase the acceptance of peripheral nerve allografts [2-4], and the immunosuppressive agent FK506 has been commonly used in nerve allotransplantation [5-9]. FK506 can enhance nerve regeneration and accelerate functional recovery not only in allograft but also in isograft- [10,11], crush- [12-14], transection- [15-17], and conduit-based animal models [18]. In a study involving neurohistomorphometric analyses on rats given daily doses of FK506 at 0.25, 0.5, 1.0, or 2.0 mg/kg, significant improvements were seen in neuroregeneration for FK506 doses of 0.5 and 1.0 mg/(kg · d) [19]. With these doses of FK506, neuroregeneration was found to be enhanced, but skin allograft rejection could not be prevented [19]. The beneficial effects of FK506 on neuroregeneration are not restricted to immediate administration, but these effects are significantly diminished when FK506 is administered at 3 d after nerve injury [17]. Currently, 3 d of pretreatment with FK506 and continued immunosuppression until the nerve growth has crossed the distal graft site are recommended on the basis of clinical results obtained for human nerve allotransplantation [20].

Sufficient immunosuppression after nerve allotransplantation is important for successful nerve regeneration through the allograft. Moreover, successful neuroregeneration is also a key factor involved in the recent emergence of composite tissue transplantation, including whole limb or facial allografts. In addition, because the peripheral nerve allografts provide sensation and function to the composite tissue allotransplants, preventing nerve allograft rejection is an important step during the transplantation of composite tissue allografts, which comprise components with different antigenicities. Tissues differ in their susceptibility to rejection [21]. The skin, lung, and small bowel are among the most susceptible to rejection; the kidney transplants show intermediate susceptibility; and the liver is one of the organs that shows lower susceptibility to rejection [22]. The nerves show intermediate susceptibility, with rejection of nerve allografts typically occurring within 7 d of transplantation in the absence of host immunosuppression [23]. For evaluation of the immunological response to the nerve allograft, different methods such as the following have been used in mice: enzyme-linked immunospot assays for INF-γ production [24,25]; mixed lymphocyte cultures [23,26]; limiting dilution analysis [23], which measures the frequency of defined clones of lymphocytes responding to a specific antigen; and neurohistomorphometric analysis. However, all these approaches require sacrifice of the animal with harvest of the spleen or nerves, which is not practical in the clinical setting. Therefore, monitoring the immunological status of the deeply buried nerve allograft without sacrificing the animal remains a challenge and requires further biomarkers that allow sensitive and accurate monitoring of graft function as well as early and specific diagnosis of rejection.

Sera and other body fluids contain cell-free DNA, RNA, and circulating nucleic acids, which serve as potential biomarkers [27]. One such biomarker that has aroused interest in recent years is a small ∼ 22-nucleotide (nt) noncoding RNA, which is called microRNA (miRNA) [28], whose specific expression patterns in the serum have been identified to contain fingerprints for various diseases [29-31]. In addition, despite the presence of ubiquitous ribonucleases (RNases), the serum miRNA levels are remarkably stable and reproducible [32,33]. Further, biochemical analyses indicate that miRNAs are resistant to RNase activity, extreme pH and temperature, extended storage, and multiple freeze-thaw cycles [34,35]. miRNA expression is tightly regulated in a tunable, cell-specific, and time-dependent manner. Moreover, secreted miRNAs offer additional advantages. First, most miRNA sequences are conserved across species. Second, the expression of some miRNAs is specific to tissues or biological stages. Third, miRNAs are active moieties and should thus reflect physiological alterations more directly than mRNAs [36]. Fourth, in contrast to the levels of protein-based biomarkers, the levels of miRNAs can be easily measured by quantitative real-time PCR (qRT-PCR), allowing for high-precision signal amplification. Moreover, the protein-based biomarkers may have different posttranslational modifications that can affect the accuracy of measurement, while miRNAs are relatively homogeneous [37]. Fifth, the use of multiple miRNA expression in a cluster pattern to analyze miRNAs in parallel increases the sensitivity and specificity and provides an accessible diagnostic tool [34,36]. This study aims to establish the expression profile of circulating miRNAs during nerve allotransplantation.

## Methods

### Experimental design

Male Balb/c and C57BL/6 mice (age, 10–12 weeks; weight, 30–35 g) were purchased from BioLasco (Yi-Lan, Taiwan). The Balb/c mice were randomized into 3 experimental groups: (1) untreated isograft, (2) untreated allograft, and (3) allograft with FK506 treatment. Additional Balb/c and C57BL/6 mice served as sciatic nerve isograft and allograft donors, respectively. These species of mice were selected on the basis of disparity at the MHC locus and prior experience with reciprocal rejection of grafts between these murine strains. FK506 was administered subcutaneously at 1 mg/(kg · d) throughout the experimental course, unless indicated otherwise. At 1, 3, 7, 10, and 14 d after initial surgery (*n* = 6 animals per group at each time point), sciatic nerve grafts were harvested, and whole blood samples were obtained. Additional sham-operated mice were subjected to the same procedure, including opening of the skin and muscle layers and exposing the sciatic nerve, but without nerve transection or nerve grafting, to measure the effect of FK506 injection on the expression of circulating miRNA. To test the effect of the discontinuation of FK506 treatment on the expression of circulating miRNA, whole blood was drawn at 0, 2, 4, and 6 d after discontinuation of FK506 injection in an additional group of mice with allografts and immunosuppression for 7 d. The whole blood samples (1 mL per mouse) were collected at the indicated times in tubes containing anticoagulant. After the whole blood samples were incubated at room temperature for 15 min, they were centrifuged at 3,000 × g for 10 min, white blood cells were slowly qRT-PCR removed from the corresponding layers, and the serum was extracted and stored at –80°C before processing for RNA analyses. All the housing conditions and the surgical procedures, analgesia, and assessments were in accordance with national and institutional guidelines, and an Association for Assessment and Accreditation of Laboratory Animal Care (AAALAC)–accredited SPF facility was used. The animal protocols were approved by the Institutional Animal Care and Use Committee (IACUC) of Kaohsiung Chang Gung Memorial Hospital.

### Surgical procedures

The mice were anesthetized by intraperitoneal injection of an anesthetic cocktail consisting of 0.1 mg/g ketamine and 0.01 mg/g xylazine. The anesthetized mice were restrained in a supine position on a heated pad to maintain the body temperature at 37°C. Under aseptic conditions, with sterile povidone/iodine preparation and 70% ethanol and sterile instruments and drapes, the skin over the proximal right hindlimb was incised, and the underlying biceps femoris muscle was bluntly dissected to expose the sciatic nerve. An established mouse sciatic nerve allotransplantation model was used. In brief, 1-cm Balb/c or C57BL/6 donor sciatic nerve grafts were transplanted in reverse orientation into 0.5-cm sciatic nerve gaps created in the recipient Balb/c mice. Tension-free repair was then performed under an operating microscope with three 11–0 nylon (Ethicon Inc., Somerville, NJ) interrupted epineurial sutures under 40× magnification. The muscle was closed with 5–0 vicryl sutures, and the skin with interrupted 5–0 nylon sutures. The animals were monitored to ensure appropriate feeding and diet after surgery.

### RNA isolation

Total RNA was extracted from the harvested nerve graft, whole blood, and serum using the mirVana miRNA Isolation Kit (Ambion, Austin, TX, USA). For the miRNA array, the purified RNA yield was determined by the absorbance at 260 nm with an SSP-3000 Nanodrop spectrophotometer (Infinigen Biotech, City of Industry, CA, USA), and RNA quality was evaluated with a Bioanalyzer 2100 system (Agilent Technologies, Palo Alto, CA, USA).

### miRNA array analysis

The Mouse & Rat miRNA OneArray® v3 (Phalanx Biotech Group, Hsinchu, Taiwan) contains a total of 4104 probes, including 144 experimental control probes, 1111 unique mouse miRNA probes, and 680 rat miRNA probes, based on miRBase version 17. Mouse genome-wide miRNA microarray analysis was performed by Phalanx Biotech. Briefly, fluorescent targets were prepared from 2.5 μg of total RNA with the miRNA ULS™ Labeling Kit (Kreatech Diagnostics, Amsterdam, the Netherlands). Labeled miRNA targets enriched using NanoSep 100K (Pall Corporation, Port Washington, NY, USA) were hybridized to the Mouse & Rat miRNA OneArray® v3 in Phalanx hybridization buffer, using the OneArray® Hybridization Chamber. After overnight hybridization at 37°C, nonspecifically bound targets were removed by 3 washing steps (wash I, at 37°C for 5 min; wash II, at 37°C for 5 min and at 25°C for 5 min; and wash III, rinse 20 times at 37°C). The slides were dried by centrifugation and scanned using an Axon 4000B scanner (Molecular Devices, Sunnyvale, CA, USA). The signal intensities of Cy5 fluorescence in each spot were analyzed using GenePix 4.1 software (Molecular Devices) and processed using the R program. We filtered out spots for which the flag was <0, and spots that passed this criteria were normalized using the 75% media scaling normalization method. Normalized spot intensities were converted into gene expression log_2_ ratios for the control and treatment groups. Spots with log_2_ ratios ≤ –1 or ≥1 and *P* values <0.05 were selected for further analysis. The differentially expressed miRNAs were subjected to hierarchical cluster analysis using average linkage and Pearson correlation as a measure of similarity.

#### Quantification of miRNA expression

miRNA expression was quantified by qRT-PCR to confirm the upregulation of expression of the miRNA targets that were detected using miRNA array analysis from the transplanted nerve grafts and whole blood of the animals in the experimental groups at 7 d after surgery. The expression of each miRNA in nerve graft and whole blood was represented relative to the expression of U6 small nuclear RNA (*U6 snRNA*), which was used as an internal control. For the serum, 25 fmol of single-stranded cel-miR-39, synthesized by Invitrogen (Invitrogen, Carlsbad, CA, USA), was spiked into 400 μL of serum as an internal control for the expression of each miRNA. In the reverse transcription step, each RNA sample was reversely transcribed to cDNA by using TaqMan® MicroRNA Reverse Transcription Kit (Applied Biosystems, Foster City, CA, USA) according to the manufacture’s instruction. In the PCR step, PCR products were mixed with the TaqMan Universal PCR Master Mix (No UNG, PN 4324018, Applied Biosystems) and specific miRNA primers from the TaqMan MicroRNA Assays (Applied Biosystems). The following TaqMan MicroRNA Assays were used in this study: mmu-miR-125b-3p (assay ID: 002378), mmu-miR-467b* (001684), mmu-miR-672 (002327) for nerve grafts and mmu-miR-320 (002277), mmu-miR-423-5p (002340), mmu-miR-762 (002028) for whole blood and serum and U6snRNA (001973), cel-miR-39 (000200) for internal control. The qRT-PCR reactions were performed by a 7500 Real time PCR system (Applied Biosystems) and each sample reactions were run in triplicate. We evaluated miRNA expression by calculating the relative expression values in 6 samples and comparing them with those from the control samples; the induction was expressed as fold change in miRNA expression relative to that for the control. Intergroup comparison was performed using analysis of variance (ANOVA) and an appropriate post hoc test to compensate for multiple comparisons (SigmaStat, Jandel, CA, USA). *P* values of <0.05 were considered significant.

## Results

### Dysregulated miRNA targets in miRNA array analysis

**Figure 1:**
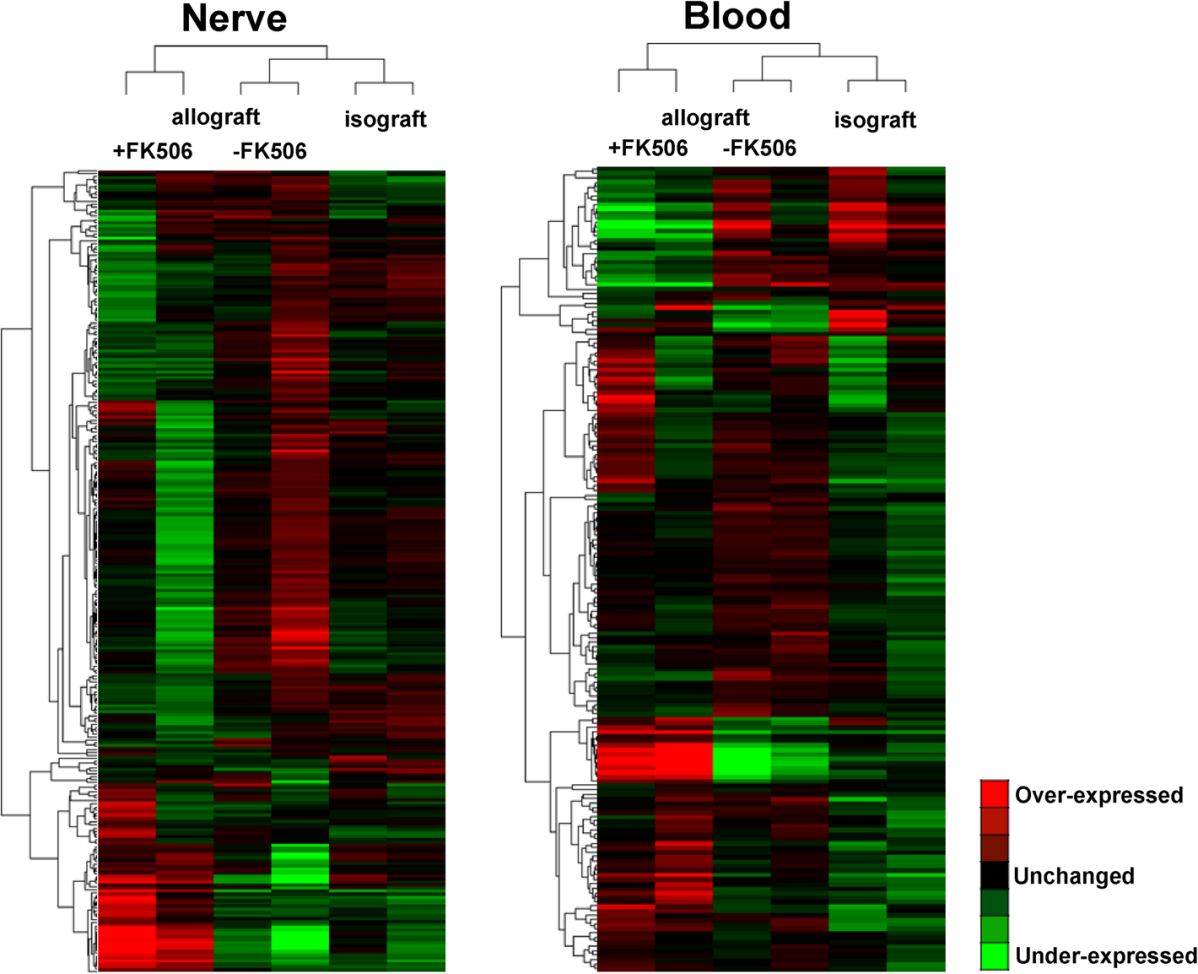
**Unsupervised hierarchical clustering of the expression of miRNAs.** Hierarchical clustering of miRNA differentially expressed in the whole blood and nerve grafts of Balb/c mice at postoperative day 7 with untreated isografts (Balb/c → Balb/c), (2) untreated allografts (C57BL/6 → Balb/c), and (3) allografts (C57BL/6 → Balb/c) with daily injection of 1 mg/kg FK506 for immunosuppression.

The miRNA array experiments showed an approximately 2-fold difference in the expression of miRNAs in the whole blood and nerve graft samples at 7 d after initial surgery for these 3 groups, that is, those with an isograft and those with an allograft with or without FK506 treatment (*P* < 0.05; *n* = 2 for each subgroup). The hierarchical cluster analysis of all miRNAs expressed in the whole blood and nerve graft is shown in Figure 1. Unsupervised hierarchical clustering was used to separate the samples from different experimental subjects into different groups. The miRNA targets that were significantly dysregulated in the array experiments are shown in Table 1. In the whole blood samples, when compared to those with untreated isograft, there were 5 (miR-720, miR-709, miR-2145, miR-1195, and miR-690) and 4 (let-7d, miR-26a, let-7i, and left-7a) downregulated miRNA targets in the mice receiving allograft without and with FK506 treatment, respectively. In addition, there was significant expression of 3 miRNAs (miR-320, miR-762, and miR-423-5p) in the mice receiving an allograft with FK506, when compared to those mice receiving allograft without FK506. When compared to mice with untreated isografts, the mice receiving allografts with or without FK506 immunosuppression did not show increased miRNA target levels. In the nerve graft, 1 miRNA (miR-125b-3p) was upregulated in the mice receiving allografts without FK506, compared to those with untreated isografts. In addition, there was significant expression of 3 miRNAs (miR-125b-3p, miR-672, and miR-467b*) in the mice receiving an allograft with FK506, when compared to those mice receiving an allograft without FK506. No increased miRNA target expression and decreased expression of only 1 miRNA target (miR-2140) were found in the mice that received an allograft with FK506 treatment and those with untreated isografts, respectively. The miRNA array data have been deposited in Gene Expression Omnibus (accession numbers: GSE48969).

**Table 1:** miRNA targets that were significantly dysregulated in the whole blood and the grafted nerves by the miRNA array experiments in the untreated isograft, untreated allograft, and allograft with FK506 immunosuppression groups

**Whole Blood**					
Allograft v.s. Sham operation		Allograft with FK506 v.s. Sham operation		Allograft with FK506 v.s. Allograft	
increased miRNA targets	log2 (ratio)	increased miRNA targets	log2 (ratio)	increased miRNA targets	log2 (ratio)
No		No		miR-320	1.44
				miR-762	1.37
				miR-423-5p	1.08
decreased miRNA targets	log2 (ratio)	decreased miRNA targets	log2 (ratio)	decreased miRNA targets	log2 (ratio)
miR-720	-1.75	let-7d	-1.43	No	
miR-709	-1.13	miR-26a	-1.42		
miR-2145	-1.03	let-7i	-1.44		
miR-1195	-1.01	let-7a	-1.25		
miR-690	-1.01				
**Nerve grafts**					
Allograft v.s. Sham operation		Allograft with FK506 v.s. Sham operation		Allograft with FK506 v.s. Allograft	
increased miRNA targets	log2 (ratio)	increased miRNA targets	log2 (ratio)	increased miRNA targets	log2 (ratio)
miR-125b-3p	1.02	No		miR-125b-3p	1.54
				miR-672	1.15
				miR-467b*	1.03
decreased miRNA targets	log2 (ratio)	decreased miRNA targets	log2 (ratio)	decreased miRNA targets	log2 (ratio)
No		miR-2140	-1.16	No	

### Profiling of miRNA expression

**Figure 2:**
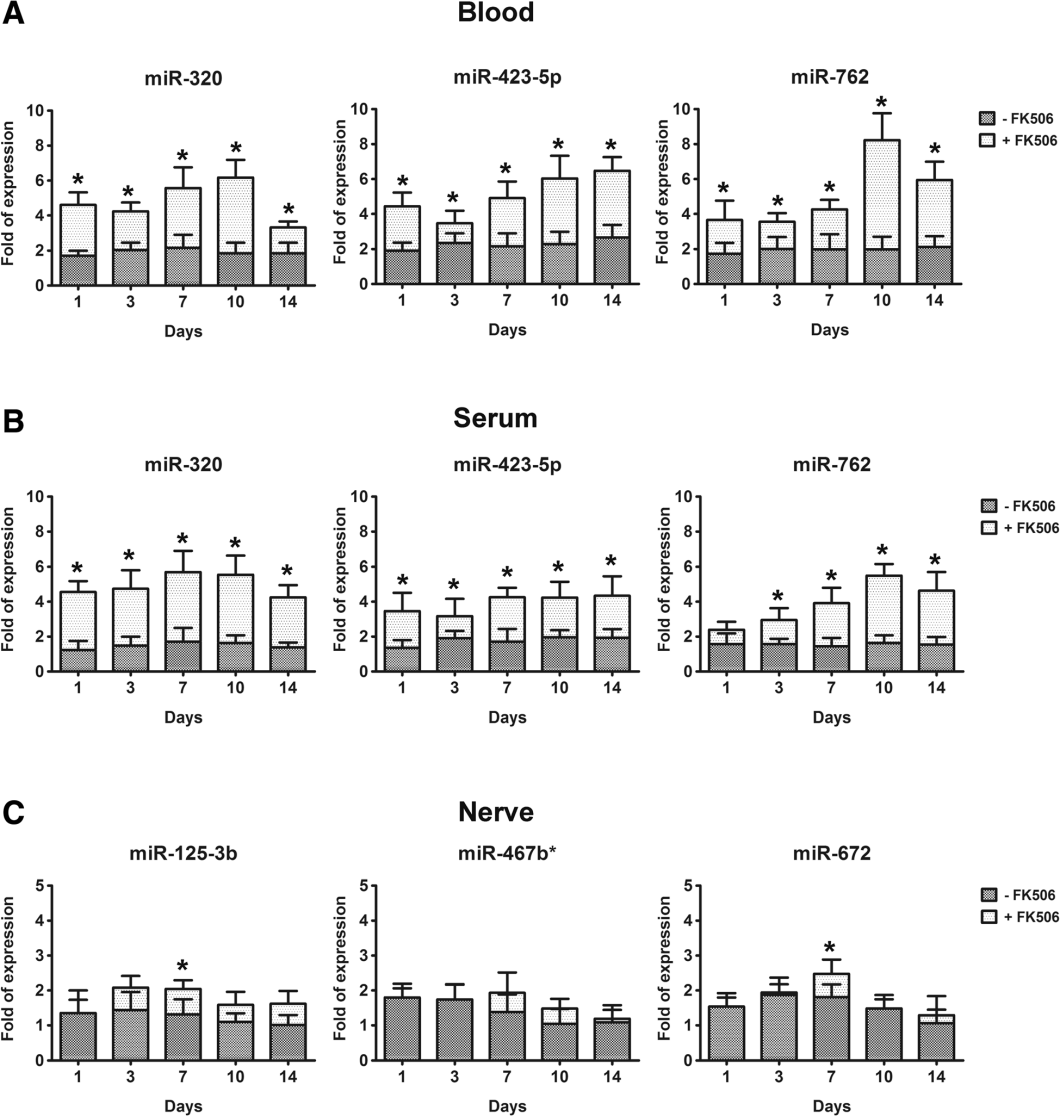
**Quantification of upregulated miRNA targets detected by miRNA arrays, by qRT-PCR within 2 weeks after nerve allotransplantation in (A) whole blood, (B) sera, and (C) nerve grafts.** Bars represent mean ± SEM values of 6 experiments; *P < 0.05.

**Figure 3:**
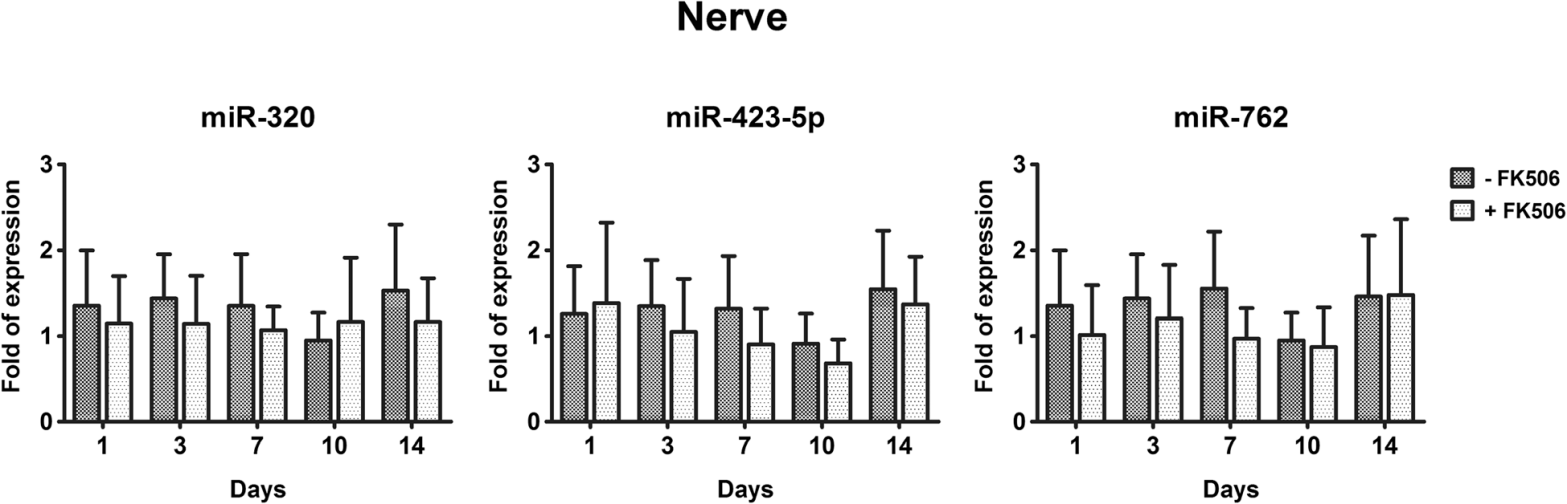
**Quantification of the circulating miRNA targets in the nerve graft within 2 weeks after nerve allotransplantation by qRT-PCR.** Bars represent mean ± SEM values of 6 experiments; *P < 0.05.

Because miRNAs are directly transcribed to cope with physiological or pathological alterations, the upregulated miRNAs were further analyzed by qRT-PCR, which was expected to give more meaningful results. Three miRNAs (miR-320, miR-762, and miR-423-5p) in the circulation and 3 miRNAs (miR-125b-3p, miR-672, and miR-467b*) in the nerve graft were subjected to further quantification and validation. Expression of the miRNAs was considered differentially regulated if the mean values for all samples (*n* = 6 in each group at each time point) demonstrated more than 2-fold difference compared with those for the control samples (*P* value <0.05) by qRT-PCR. With around 4- to 6-fold expression, miR-320, miR-762, and miR-423-5p were significantly upregulated in the whole blood of the mice receiving allograft with FK506 at 1, 3, 7, 10, and 14 d after nerve allotransplantation, when compared to those mice receiving allograft without FK506 (Figure 2A). The expression of these 3 miRNA targets (miR-320, miR-762, and miR-423-5p) was found to be significantly upregulated in the serum (Figure 2B). In the nerve graft, only miR-125-3b and miR-672 were significantly upregulated in the mice receiving allograft with FK506 only at 7 d, but not at 1, 3, 10, or 14 d, after nerve allotransplantation (Figure 2C). However, the increase of miR-125-3b and miR-672 at 7 d was only a little, albeit significantly. No significant expression of miR-467b* was noted in the nerve graft within 2 weeks after nerve allotransplantation. In addition, no significant expression of these 3 circulating miRNAs (miR-320, miR-762, and miR-423-5p) was found in the nerve graft of the mice receiving allograft with FK506 (Figure 3).

### Expression of circulating miRNAs after discontinuation of FK506

**Figure 4:**
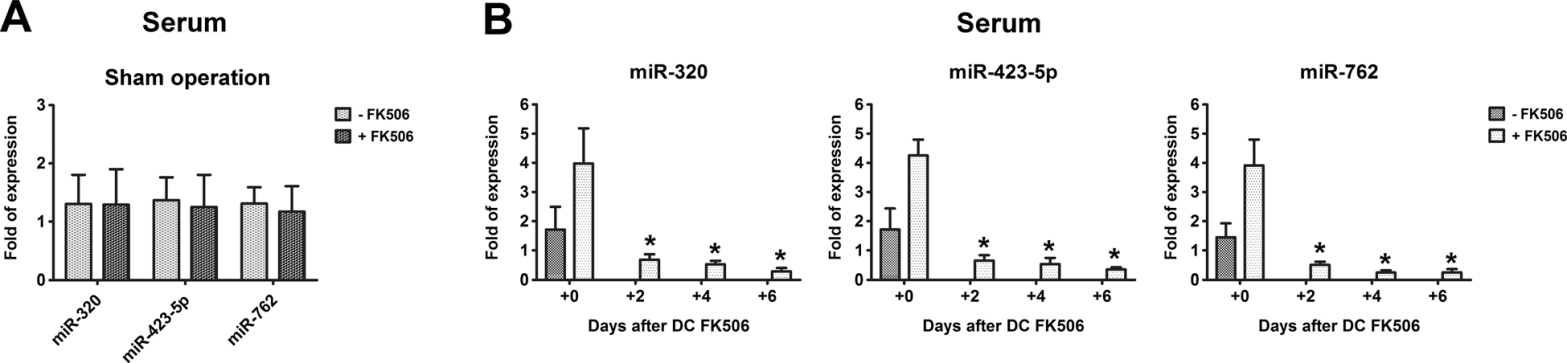
**Quantification of the circulating miRNA targets by qRT-PCR in (A) the serum drawn from sham-operated mice at day 7 after nerve allotransplantation with or without daily FK506 injection and (B) at 2, 4, and 6 d after discontinuation of FK506 injection in the mice receiving nerve allotransplantation with FK506 immunosuppression for 7 d.** Bars represent mean ± SEM values of 6 experiments; *P < 0.05.

To clarify whether the expression of circulating miRNAs was induced by FK506 per se, that is, regardless of the nerve allotransplantation, we quantified the expression of miR-320, miR-762, and miR-423-5p in the serum drawn from sham-operated mice with or without daily FK506 injection for 7 d by using qRT-PCR. The results revealed no significant difference between these 2 groups (Figure 4A). In addition, in the Balb/c mice with allograft and FK506 immunosuppression, the expression of all the 3 upregulated circulating miRNAs showed a significant decrease at 2, 4, and 6 d after discontinuation of FK506 injection; the expression was even markedly lower than that in the mice at 7 d after untreated nerve allotransplantation.

## Discussion

In this study, we established the profile of circulating miRNA expression using a murine model of nerve allotransplantation. miR-320, miR-762, and miR-423-5p were significantly upregulated in the whole blood and serum, but not in the nerve graft, of the mice receiving an allograft with FK506 immunosuppression at 1, 3, 7, 10, and 14 d after nerve allotransplantation. All these 3 upregulated circulating miRNAs showed a significant decrease at 2, 4, and 6 d after discontinuation of FK506 injection.

The introduction of powerful immunosuppressive therapies in the past 4 decades has reduced the incidence of acute rejection in transplant recipients. However, the lack of noninvasive biomarkers of rejection precludes optimization of therapeutic intervention. Allograft biopsy is the gold standard for diagnosis of conditions like acute rejection, disease recurrence, and drug toxicity [38,39]. However, whereas minimal risk is involved in obtaining an allograft biopsy of solid organs, it is not practical to acquire a nerve biopsy in the clinical setting. Moreover, the histological evaluation of biopsies remains subjective and is associated with some degree of variability depending on the pathologist evaluating the tissue sample [39-41]. Circulating miRNAs, owing to the noninvasive nature of their detection, their disease specificity, and the availability of accurate techniques for their detection and monitoring, may be developed as excellent biomarkers of allograft injury and function [42]. Sui et al. reported the first comparison using microarray analysis of miRNA expression and qRT-PCR confirmation in acute rejection after renal transplantation and identified 8 upregulated and 12 downregulated miRNAs differentially expressed in acute rejection after renal transplantation [43]. Anglicheau et al. showed a set of miRNAs to be highly deregulated in renal biopsy samples and peripheral blood mononuclear cells of patients with acute rejection [44]. Intragraft levels of miR-142-5p, miR-155, miR-223, miR-10b, miR-30a-3p, and let-7c have been proposed to have diagnostic value for acute rejection, with miR-142-5p, miR-155, and miR-223 each predicting acute rejection with >90% sensitivity and specificity. Upregulated miR-142-5p, miR-155, and miR-223 were strongly linked to intragraft levels of CD3 and CD20 mRNA, suggesting that the altered expression of miRNAs might be due to graft-infiltrating immune cells [44]. Asaoka et al. proposed that the different miRNA expression patterns can be used to identify novel biomarkers and therapeutic targets for immunosuppressive agents; their study involved evaluation of the expression of 384 mature miRNAs in intestinal mucosal biopsy specimens from recipients with small bowel transplantation [45]. Recently, Lorenzen et al. found the urinary miR-210 to be the only specific urinary biomarker of acute cellular rejection in transplant patients [46]. Furthermore, differential expression of miR-142-3p, miR-204, and miR-211 was also observed in urine samples between patient groups with chronic allograft dysfunction [47]. Gehrau et al. also reported that the abnormal levels of circulating cell-free miRNAs correlate with specific hepatic injury and thus may serve as feasible monitoring and outcome predictive biomarkers in liver transplantation [48]. Despite the few studies published in the field regarding the relationship between circulating miRNAs and status of allograft, the potential of miRNAs as biomarkers for diagnosis of acute or chronic rejection and the response to therapy in noninvasive monitoring might be expected to have critical impact in the transplant field in the immediate future.

Among the 3 upregulated circulating miRNAs, miR-320 was reported to increase more than 2.5-fold in neurodegeneration [49]. After injury, miR-320 might enhance neuronal regeneration and play a role in neuronal development [50]. In Neuro-2A cells, the increase in the levels of miR-320 for 3 d markedly increased neurite length, with possible involvement of the identified miR-320 targets cAMP-regulated phosphoprotein 19 kDa (ARPP-19) and semaphorin 3A detected by in silico analysis [50]. In addition, the neural precursor cell-enriched miR-762 could translationally downregulate adenosyl methionine decarboxylase 1 (Amd1), a key enzyme in the polyamine synthesis pathway, to regulate both embryonic stem cell self-renewal and differentiation into neural lineage [51]. miR-762 has also been reported to negatively regulate the innate defense genes RNase7, ST2, and Rab5a [52]. Elevated serum levels of miR-423-5p correlated with important clinical prognostic parameters in systolic heart failure patients [53] and served as a diagnostic biomarker for heart failure caused by dilated cardiomyopathy [54] or left ventricle remodeling after myocardial infarction [55]. miR-423 has been defined also as a new oncogenic miRNA in hepatocellular carcinoma through the suppression of the tumor suppressor p21Cip1/Waf1 [56]. No association of miR-423-5p with neurological illness or immunological condition has been reported in the literature.

The underlying mechanistic basis for circulating miRNA expression during an episode of acute rejection remains an area of utmost interest, which is just in its early stages of research. In this study, our main goal is to find the possible upregulated circulating miRNAs after nerve allotransplantation in the mice without immunosuppression, and then determine whether these identified circulating miRNAs may be used as targets for monitoring or therapeutic intervention. However, when compared to those with untreated isograft, no increased miRNA target was found in the circulation of the mice receiving allograft with or without FK506 treatment. We only found 3 significantly expressed circulating miRNAs (miR-320, miR-762, and miR-423-5p) in the mice receiving allograft with FK506 against those without FK506 immunosuppression. However, in this study, daily FK506 injection for 7 d in sham-operated mice revealed no significant increase of these 3 circulating miRNAs in the serum, which implies that the expression of circulating miR-320, miR-762, and miR-423-5p can only be induced under FK506 immunosuppression, but not under allogeneic condition, and therefore may potentially serve as biomarkers for sufficient immunosuppression. In addition, the immunosuppressive drug FK506 has been proved to have neuroprotective and neurotrophic actions in experimental models, that is, it increases neurite elongation and accelerates the rate of nerve regeneration in vitro and in vivo [57,58]. Furthermore, recent studies have suggested that the differentiation of different subsets of CD4 T cells is regulated by the signals present in the tissue microenvironment (Lal et al., 2011; Gao et al., 2012) and that miRNAs play an important role in the development and function of all subsets of CD4 T cells (Sethi et al., 2013). miRNAs are differentially expressed between the mice receiving allogeneic grafts with and without immunosuppression, suggesting their potentially important regulatory functions in graft rejection. Whether these circulating miRNAs play a role in the nerve regeneration-enhancing activity of FK506 requires further investigation.

There are certain limitations to our study. First, molecular insights into the origins as well as mechanisms underlying the dysregulation of circulating miRNAs could not be provided. In this study, we have demonstrated that none of the 3 circulating miRNAs (miR-320, miR-762, and miR-423-5p) was significantly expressed in nerve allografts; effective models for identifying the origin of these expressed circulating miRNAs are still not available. Second, although FK506 administered subcutaneously at 1 mg/(kg · d) is a standard and effective protocol for immunosuppression after nerve allotransplantation in rodents and in humans [17,20,24] and we have also demonstrated that discontinuation of FK506 injection will markedly downregulate these circulating miRNAs at 2, 4, and 6 d and thereafter following discontinuation, the response of these expressed circulating miRNAs to the conditions of under- or over-immunosuppression or even to organ toxicity remains obscure. Third, in the rejection of nerve allografts typically occurring within 7 d of transplantation in the absence of host immunosuppression [23], although an abrupt discontinuation of FK506 injection is expected to induce an ongoing acute rejection of the nerve allograft, there is still a time discrepancy between the discontinuation of immunosuppression and the beginning of rejection. The relationship between the decreased expression of these circulating miRNAs after the discontinuation of immunosuppression and the beginning of rejection is yet to be defined. Finally, the impact on the circulating miRNAs expression by nerve regeneration acrossing the nerve allograft should be considered and clarified in further experiment.

## Conclusions

In conclusion, this study provides the first insights into the expression of circulating miRNAs in nerve allotransplantation with or without FK506 immunosuppression. We identified the circulating miR-320, miR-762, and miR-423-5p as potential biomarkers for monitoring the immunosuppression status of nerve allografts. However, further research is required to evaluate the prognostic value of these markers and to expand the miRNA signature for disease progression.

## Competing interests

The authors declare no potential conflict of interests.

## Authors’ contributions

CSR, JCY, and SCW contributed to analysis and acquisition of all data and the writing of the manuscript. YCC participated in qRT-PCR experiment. THL, YCW and SLT participated in the animal surgery and acquisition of the study specimens. MWL participated in analysis of all data. CHH contributed to the design of animal study, interpretation of the analyzed results and the writing of the manuscript. All authors read and approved the final manuscript.
